# Oxidative Stress as a Potential Mechanistic Bridge Between Electronic Cigarette Components and Chronic Disease: A Combined Narrative and Bibliometric Analysis

**DOI:** 10.3390/antiox15080972

**Published:** 2026-08-05

**Authors:** Rana Abdel Sater, Youssef El Rayess, Sofi G. Julien

**Affiliations:** 1 Department of Nutrition and Food Sciences, Faculty of Arts and Sciences, Holy Spirit University of Kaslik, Jounieh P.O. Box 446, Lebanon; rana.f.abdelsater@net.usek.edu.lb; 2 Department of Agriculture and Food Engineering, School of Engineering, Holy Spirit University of Kaslik, Jounieh P.O. Box 446, Lebanon; youssefrayess@usek.edu.lb

**Keywords:** electronic cigarette, vaping, aerosol, oxidative stress, e-cigarette regulations, nicotine, chronic diseases, bibliometric analysis, public health

## Abstract

Despite their introduction two decades ago as cessation tools, vaping devices and electronic cigarettes (ECs) have surged dramatically among adolescents and adults worldwide. The widespread misconception that ECs are harmless has raised substantial concern about their potential contribution to chronic diseases associated with oxidative stress (OS) pathways. To map this rapidly growing field, we combined a bibliometric analysis of human research on vaping-associated OS over the past decade with a narrative review of EC component toxicology and regulatory updates. The bibliometric analysis of 189 human studies identified four keyword clusters reflecting clinico-epidemiological, biological/mechanistic, methodological, and acute-event research themes. The results of this bibliometric analysis structure the narrative synthesis, which evaluates the chemical constituents of ECs, including e-liquid constituents, device-derived contaminants, and their aerosol by-products, and links them to pathways associated with OS development. Oxidative stress was the most frequent bridging keyword, reflecting predominant academic trends as research shifted from acute-focused investigations toward mechanistic chronic disease research. No prior review has integrated EC component toxicology with bibliometric evidence to map this landscape. This combined approach provides researchers, clinicians, and policymakers with an integrated evidence base and identifies priority areas for future investigation, including long-term exposure thresholds, age- and sex-specific outcomes, and the urgent need for harmonized global regulation.

## 1. Introduction

The credit for the first electronic smoking device is attributed to Herbert A. Gilbert, who was acutely aware of the health consequences of tobacco smoking in the early 1960s. Ahead of his time, Gilbert described a “smokeless non-tobacco cigarette” that “heated moist, flavored air” [1] that no manufacturer was prepared to commercialize, a concept at that time. Forty years later, the Chinese pharmacist Hon Lik brought the electronic cigarette (EC) to market as an “electronic atomization cigarette,” explicitly designed to reduce nicotine consumption and assist with smoking cessation [2,3]. ECs are built on four core components: (1) a mouthpiece, (2) a cartridge, pod, or tank containing the e-liquid, (3) a heating element or atomizer, and (4) a rechargeable battery. Each of these constituents, particularly the metallic heating elements, can release compounds under certain conditions of use; therefore, any associated health risk is a function of exposure rather than the mere presence of the material. The operating principle of ECs involves heating the e-liquid at temperatures ranging from 100 °C to 315 °C through a metal coil, generating a vapor or aerosol that is inhaled by the user [4]. Unlike conventional cigarettes, ECs do not generate sidestream smoke but instead produce an exhaled aerosol, whose composition and concentration are influenced by respiratory retention and dilution in ambient air [5,6].

The chemical transformation of e-liquid components into aerosol-containing solid particles has raised substantial global health concerns. E-liquids consist principally of propylene glycol, glycerol, flavorings, and, in many but not all products, nicotine. During heating, these components can undergo thermal degradation to byproducts such as formaldehyde, acrolein, and acetaldehyde [7,8]. Although ECs do not generate the tar and carbon monoxide associated with conventional tobacco cigarettes, the chronic health consequences of aerosol inhalation remain an area of intensive investigation aimed at establishing global safety, toxicological, and regulatory standards.

The prevalence of vaping is now high among young people who have never smoked [9] and societal trends have distorted the original cessation rationale behind EC design, shifting its predominant use away from the original harm-reduction rationale toward a broader public health concern among never-smokers [10]. Discrepancies between declared and actual nicotine levels in e-liquids compound this problem. Indeed, products advertised as nicotine-free frequently contain measurable and even high levels of tobacco-derived or synthetic nicotine [11,12]. Motivations for EC use are multifactorial and include social acceptability, cost-effectiveness, the appeal of novel flavors, and the misperception of reduced harm relative to traditional cigarettes [13]. In interpreting these trends, it is important to distinguish the absolute risk of EC use among non-smokers and adolescents from the relative risk among adult smokers who switch completely from traditional cigarettes to ECs. Only a limited number of organizations have comprehensively documented countries that have implemented EC regulations [14,15], and elaborating a global policy consensus on preventing youth use remains an ongoing challenge [16]. Indeed, this concern is especially acute for adolescents and young non-smokers. As recently reviewed, ECs, which represent complex chemical emission sources, have been characterized as “wolves in sheep’s clothing” [17], reflecting that the diversity of chemical components and flavors they can deliver makes these devices far more than simple nicotine vehicles [18]. This framing applies to absolute risk in never-smokers and adolescents and is distinct from the question of relative benefits among adult smokers who switch completely from traditional cigarettes [19].

Understanding the toxicological consequences of chronic EC use is therefore essential, as aerosol-contained substances—metals, metalloids, carbonyl compounds, and reactive oxygen species (ROS)—adversely affect multiple physiological systems, particularly the respiratory, cardiovascular, and digestive tracts [20].

Despite this extensive background on electronic cigarettes and chronic disease, no prior review has simultaneously synthesized the chemical toxicology of EC components from a mechanistic perspective while systematically mapping the structure, scope, and thematic evolution of that literature. This review addresses this gap through two complementary approaches: (1) a bibliometric analysis that quantitatively characterizes the global landscape of vaping-associated OS in human populations over the past decade, revealing the global research landscape, thematic clusters, and knowledge gaps; and (2) a narrative synthesis structured around these bibliometric findings evaluates the latest evidence on EC chemical components, aerosol toxicology, and pathways associated with OS development underlying the clinical clusters observed in the bibliometric analysis. Together, these approaches provide researchers, clinicians, and policymakers with an integrated, evidence-based roadmap of the field.

## 2. Bibliometric Landscape of Vaping and Chronic Disease Research (2016–2026)

### 2.1. Bibliometric Methods

#### 2.1.1. Data Selection and Search Strategy

**Figure 1:**
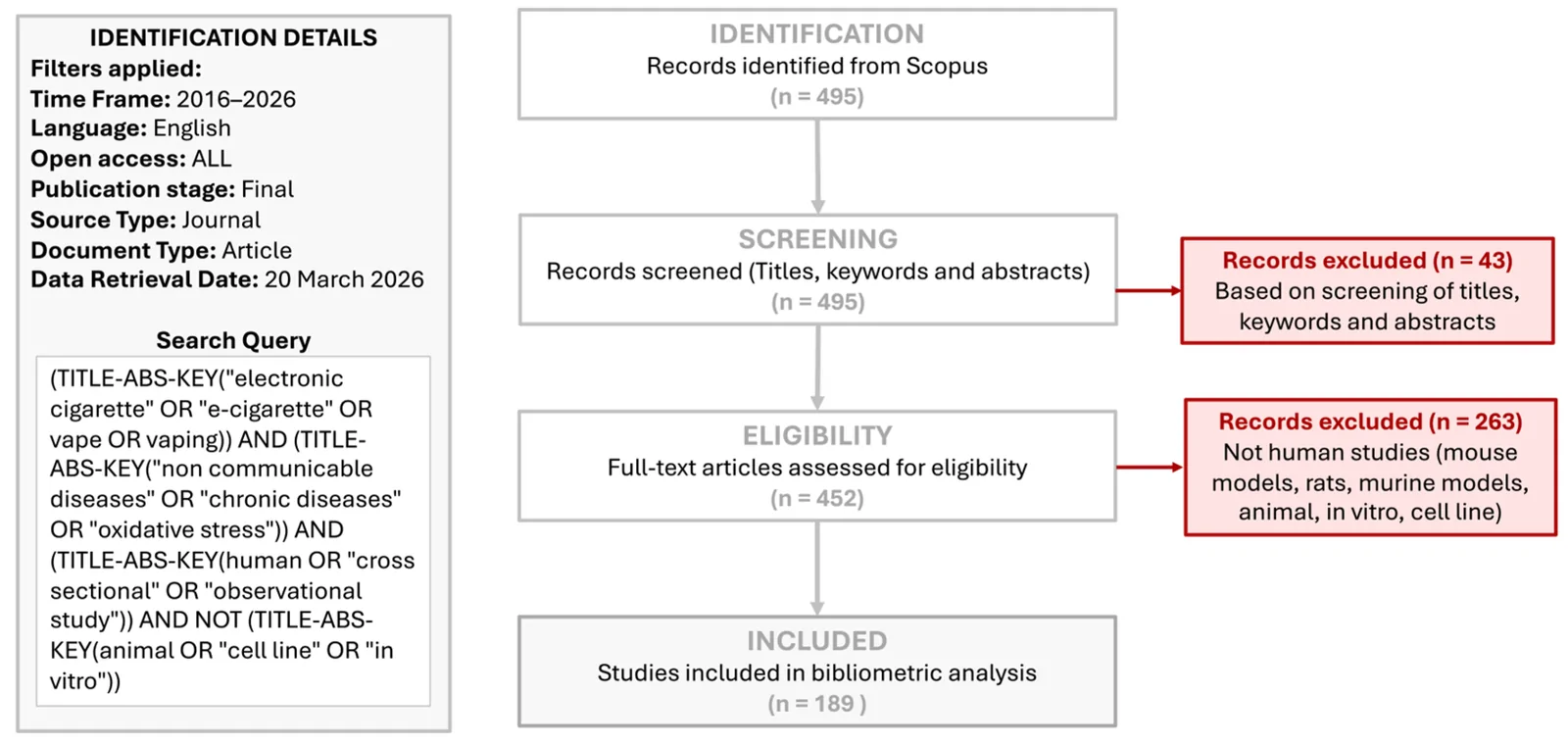
Flow diagram of the literature search and selection process for bibliometric analysis.

The comprehensive bibliometric search was conducted in the Scopus database on 20 March 2026, using a targeted query combining terms for electronic cigarettes, chronic diseases, and human studies, while explicitly excluding in vitro and in vivo model studies (Figure 1). Scopus was selected for its broad coverage of biomedical journals, modern data volume, and standardized affiliation and keyword indexing. The search was restricted to a 10-year publication window (2016–2026), peer-reviewed journal articles at the final publication stage, and English-language documents, yielding 495 initial records.

#### 2.1.2. Exclusion and Inclusion Criteria

Title, abstract, and keyword screening led to the immediate exclusion of 43 irrelevant documents. Full-text evaluation during the eligibility phase eliminated an additional 263 studies due to duplication, a lack of thematic relevance, or non-human study methodology. Ultimately, 189 publications met all predefined criteria and were included in the final bibliometric synthesis strictly limited to human clinical and epidemiological studies.

#### 2.1.3. Data Visualization

Bibliometric data were visualized using the Biblioshiny interface within the R package ‘bibliometrix’ (R Project 4.5.3), VOSviewer (version 1.6.19) and Mermaid Live Editor. Visualizations include annual production curves, geographic distribution maps, journal source analyses, cited document analyses, keyword word clouds, trend topic charts, and co-occurrence networks. Keyword analyses were performed on Scopus-indexed keywords, which combine author keywords with controlled descriptors assigned by the database. No thesaurus file or stop-list was applied; synonyms were not harmonized, and all generic indexing descriptors are reported.

### 2.2. General Research Growth and Geographic Landscape

**Figure 2:**
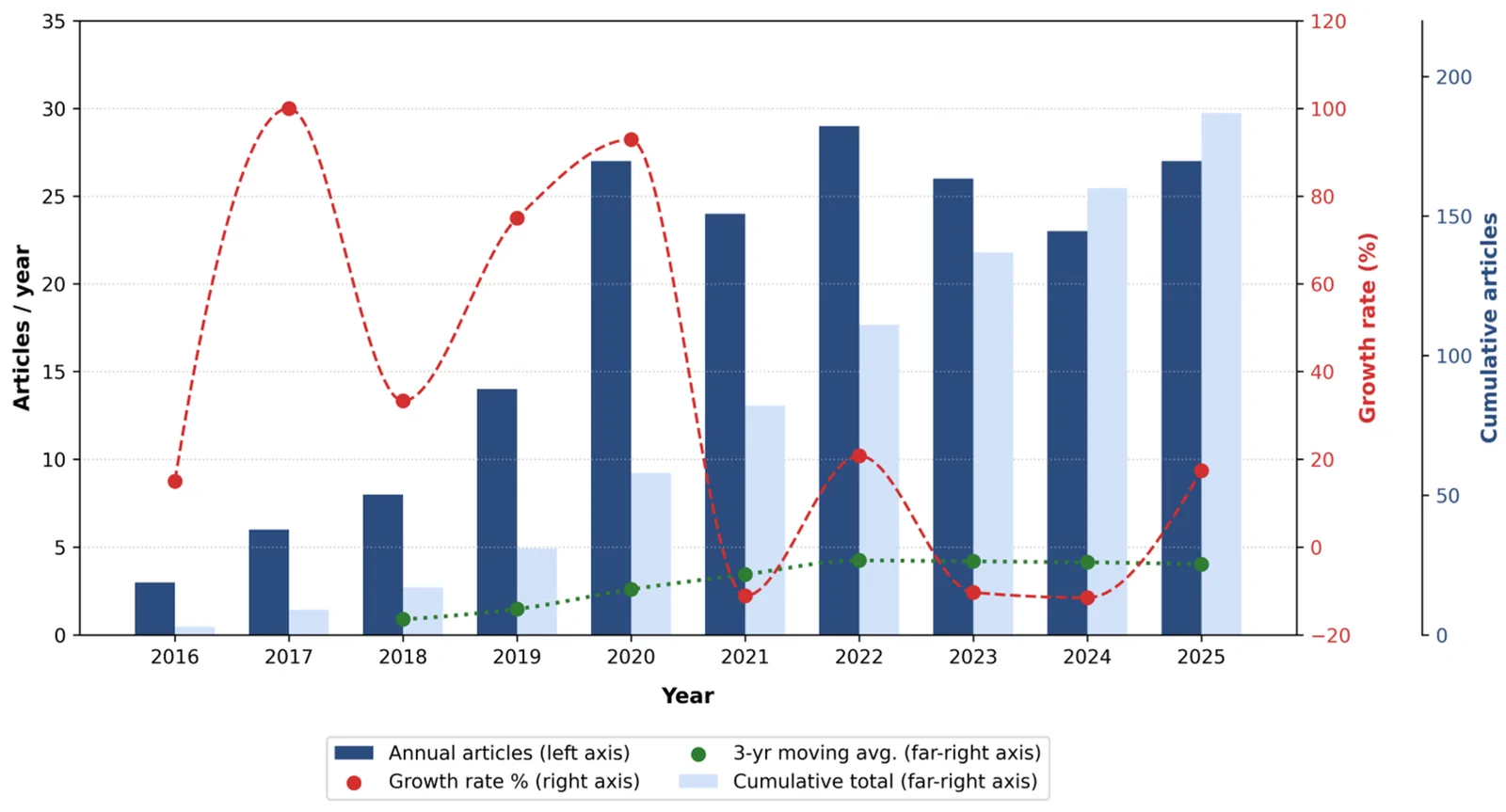
Annual scientific production on OS-associated chronic disease from vaping (Scopus, 2016–2026).

The annual scientific production in the field of OS-associated disease from vaping shows two distinct phases, as illustrated in Figure 2. Phase 1 is marked by steady and rapid growth, with a productivity of 3 articles in 2016 to a peak of 27 articles in 2020. Phase 2 is marked by stabilization, with annual publications fluctuating between 24 and 28 articles between 2020 and 2025, and a sharp decline in growth rates. The transition from Phase 1 to Phase 2 coincides with the EVALI outbreak that triggered global interest in this research, which remained active but not exponential. A 3-year moving average indicates that the apparent decline in 2021 was not a true deceleration but instead a normal inter-annual variation. This pattern demonstrates a classic lifecycle observed in bibliometric analyses, where the focus progresses from the identification of a global health burden to achieving scientific recognition.

### 2.3. Geographic Landscape, Contributing Countries and Institutions

**Figure 3:**
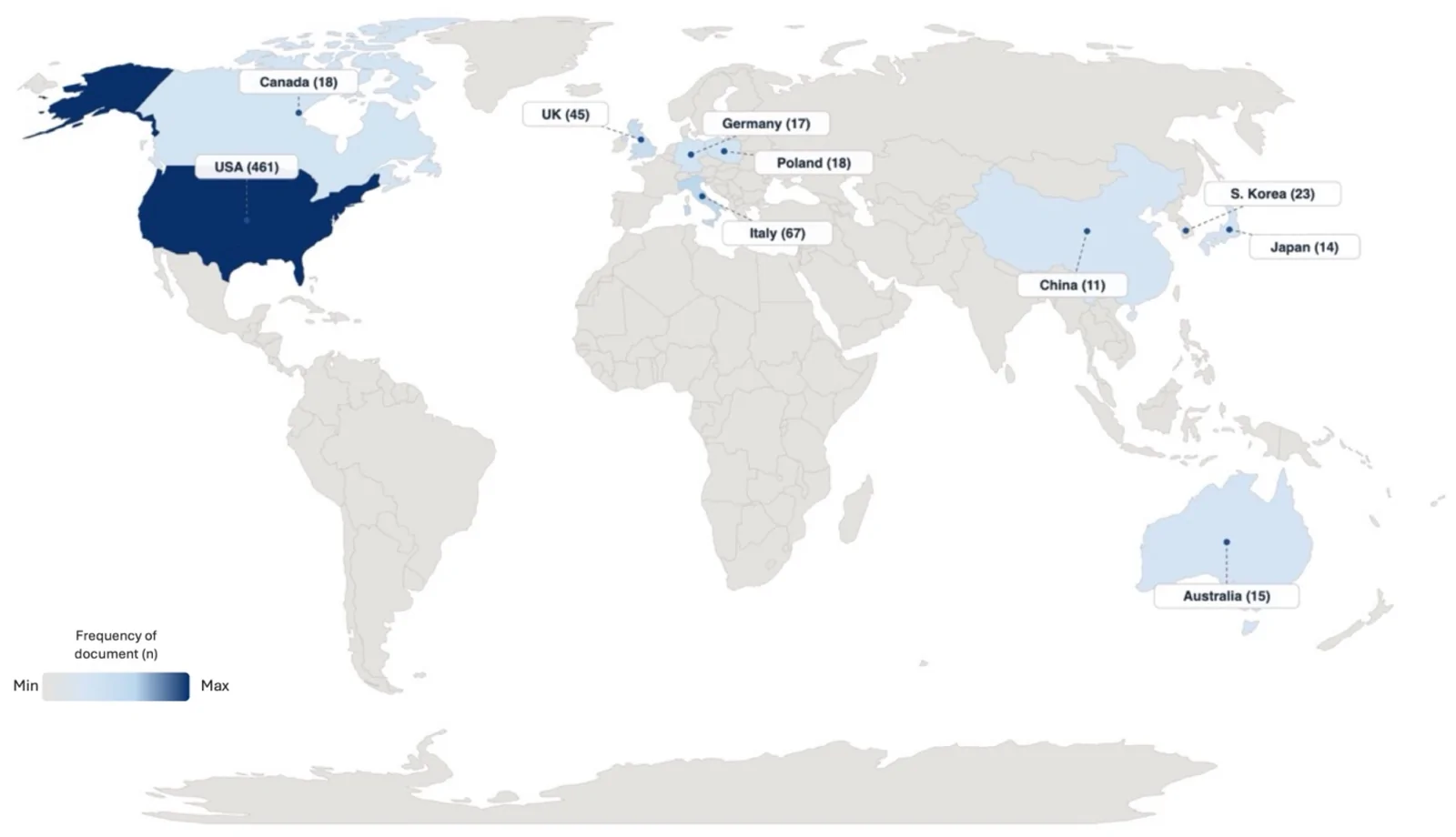
World choropleth map of scientific production by country (2016–2025).

**Figure 4:**
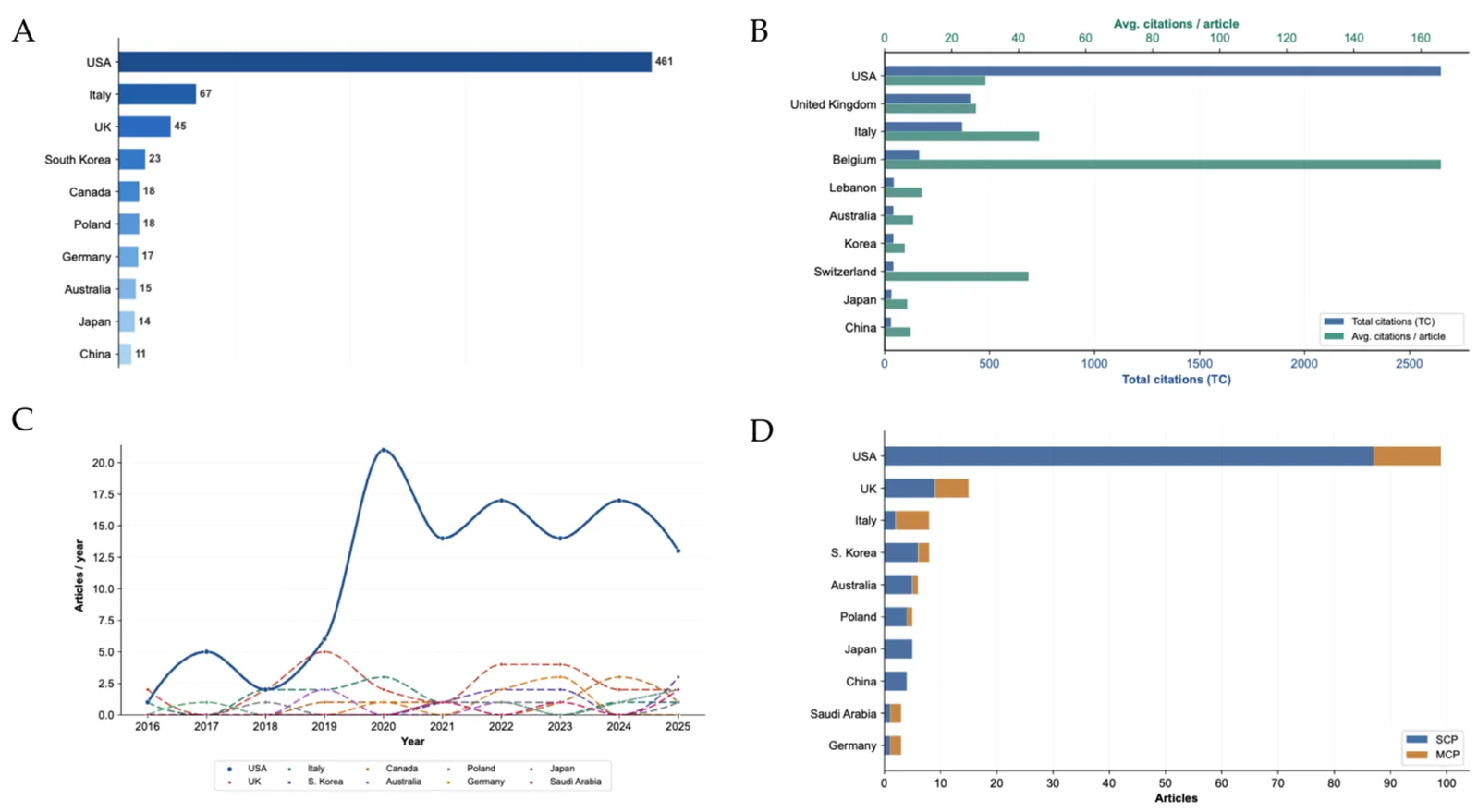
Scientific production on OS-associated disease from vaping. (**A**) Top 10 countries by country-affiliation occurrences; (**B**) Total citations (TC) and average citations per article for the 10 most cited countries; (**C**) Country Scientific production over time; (**D**) Single-country publication (SCP) and multiple-country publication for the 10 leading corresponding-authors countries (MCP).

As shown in Figure 3 and Figure 4A, the United States (US) largely dominates the global output of research on vaping-associated OS with 461 country-affiliation occurrences. This metric represents the number of times the country appears among author affiliations, which can exceed the number of articles because a single article may list authors affiliated with several countries. The US is followed by Italy with 67, the United Kingdom (UK) with 45, and South Korea with 23 country-affiliation occurrences. Although the US also leads with a total of 2650 citations and an average of 30.1 citations per article, Italy has the highest average of 46.2 citations per article. Belgium achieves nearly the same total citation average per article while having far fewer articles than the US (Figure 4B). Surprisingly, Japan and China, which are usually among the world’s top research producers, do not show the expected ratios based on their recognized research capacity in this field. This may be due to an Anglo-Saxon language bias, indexing bias, or an artifact in the database used. Notably, Lebanon’s fifth position suggests that the country has a small output (4 articles) but a notable research impact with an average of 11.2 citations per article from a total of 45 citations. This suggests that research impact is not necessarily a function of a country’s resources or size. The US also ranks first for publication output over time for the selected period, with a notable surge after 2019 (Figure 4C), but has the lowest level of international collaboration compared to other countries (Figure 4D). However, the overall SCP/MCP analysis shows that despite the global health burden associated with vaping, research remains predominantly intra-country and siloed, with a very low level of international collaboration reflected by the multi-country publications (MCP). This observation explains why the ten top contributing institutions in the field of OS-associated vaping disease are American. These are led by the University of California, followed almost equally by the CDC’s National Center for Environmental Health and the FDA’s Center for Tobacco Products (FDA) (Table 1). Their leading role is expected as they are the main regulatory bodies for tobacco marketing controls, toxic exposure, and non-infectious environmental hazard surveillance. In contrast, Italy and the UK show a high MCP fraction consistent with their extensive European collaborative network.

### 2.4. Most Relevant Sources and Authors

**Figure 5:**
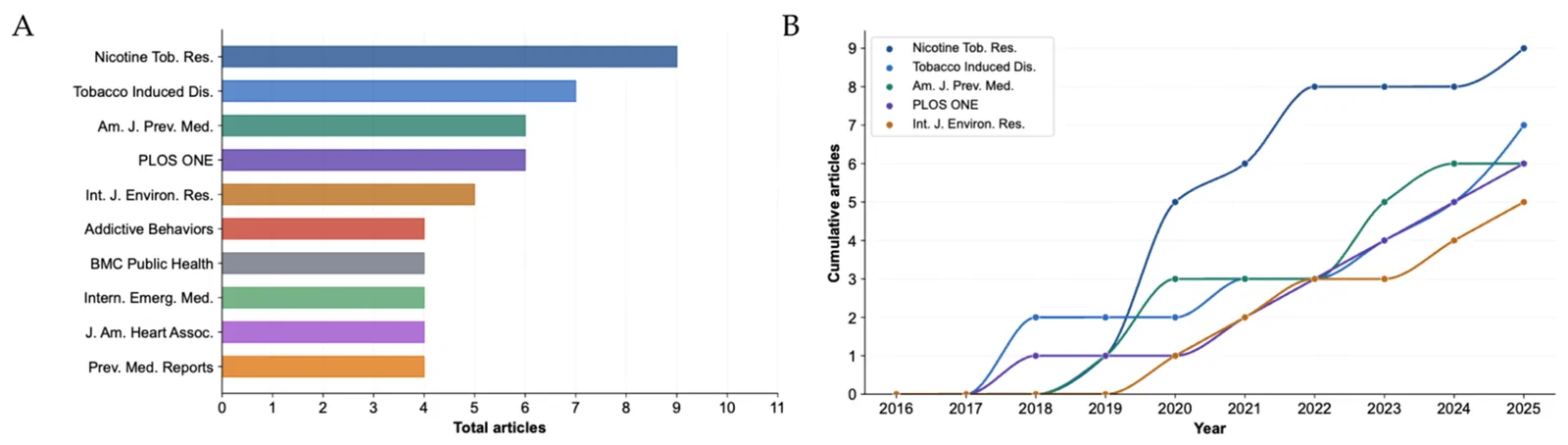
Publication sources. (**A**) Top 10 journals by article count; (**B**) Cumulative journal publication over time (2016–2025).

Nicotine and Tobacco Research and Tobacco Induced Disease rank first among the 10 top journals publishing on vaping-associated OS and chronic diseases. They are followed by interdisciplinary journals such as the American Journal of Preventive Medicine and PLOS ONE (Figure 5A). These sources demonstrate consistent cumulative publication growth, especially from 2019 onward for Nicotine and Tobacco Research and notably the International Journal of Environmental Research in line with the EVALI outbreak (Figure 5B). The multi-scope pattern of these sources reflects the multidimensional nature and broad spectrum of the vaping-related health outcomes.

**Figure 6:**
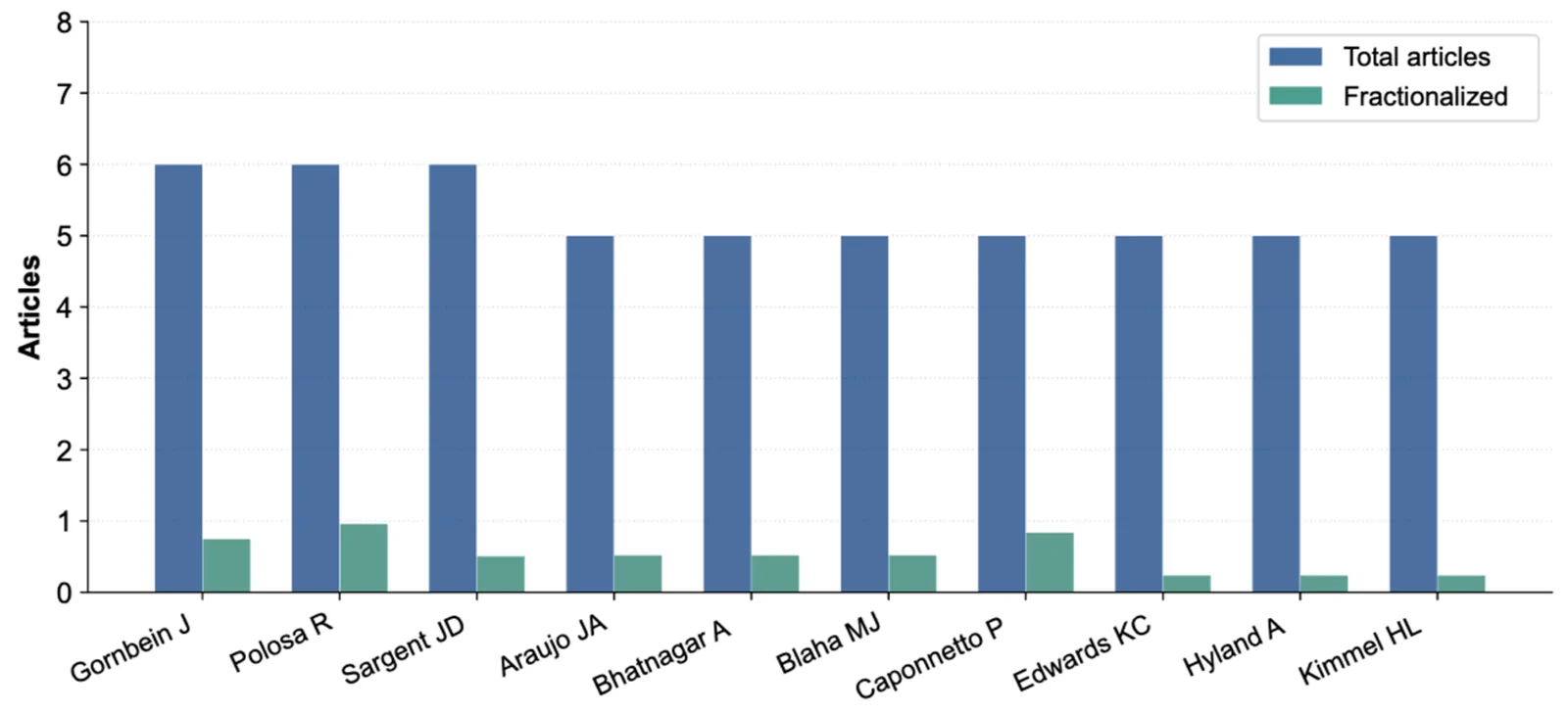
Ten most productive authors by article count (blue) and fractionalized count (teal) side by side.

**Figure 7:**
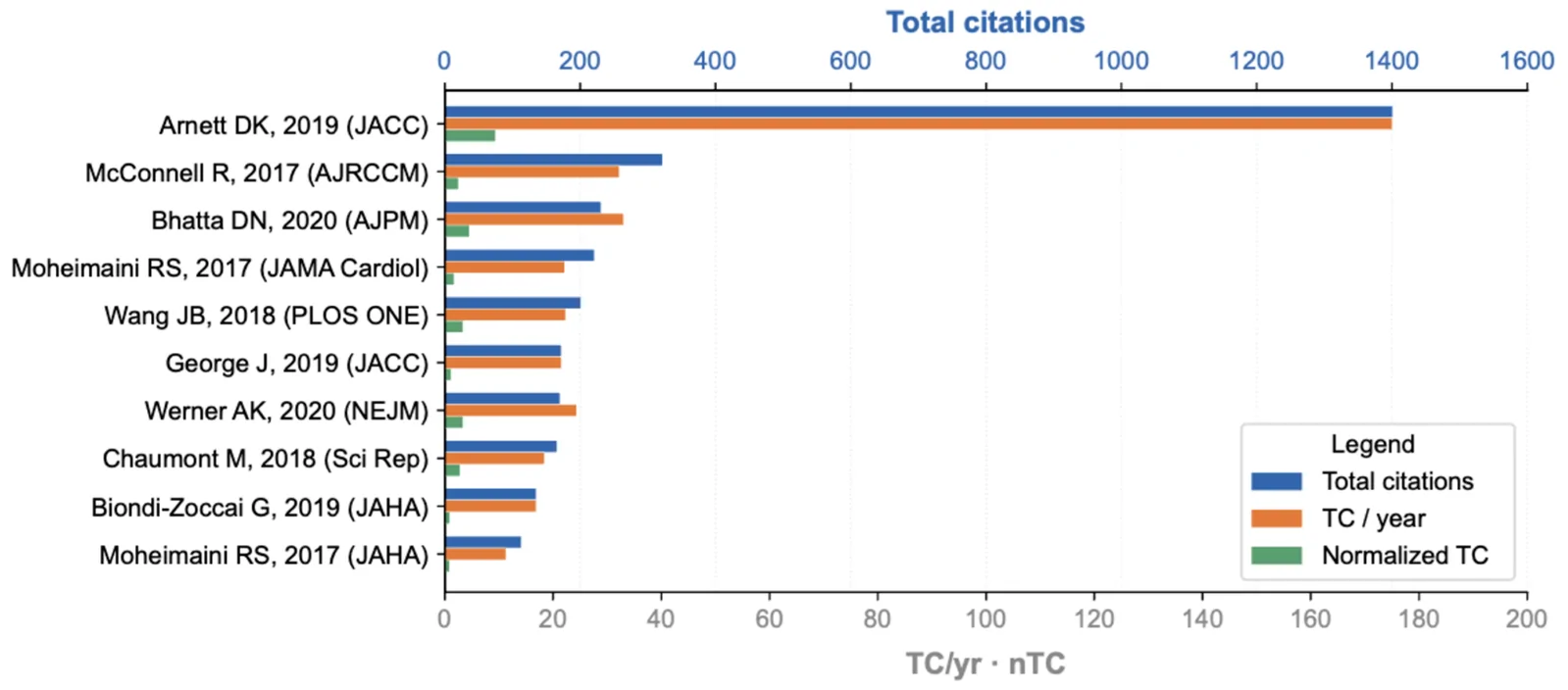
Ten most globally cited documents ranked by total citation count [21,22,23,24,25,26,27,28,29,30].

Figure 6 shows a tie among Gornbein J, Polosa R and Sargent JD, the most productive authors in the field with 6 articles each, followed closely by seven authors with 5 articles each. This distribution suggests that vaping-associated cardiovascular and respiratory disease research is a highly competitive domain. Fractionalized scores below 1 for all these authors highlight the abundant co-authorship, a common trait in clinical and epidemiological research fields. Interestingly, these authors were not among the contributors of the most cited articles (Figure 7). Indeed, the most cited article by far, with 1437 citations, is by Arnett DK et al., published in 2019 in the Journal of the American College of Cardiology (JACC) [21]. This work is the 2019 ACC/AHA Guideline on the Primary Prevention of Cardiovascular Disease. It provides a general cardiovascular prevention guideline rather than a guideline specific to vaping, which may explain its high overall citation count. The next two most cited articles are by McConnell R et al. (2017, Am J Respir Crit Care Med, TC = 322) and Bhatta DN et al. (2020, Am J Prev Med, TC = 231) [22,23], addressing vaping-associated pulmonary disease in adolescents and cardiovascular disease at the population level, respectively. The normalized total citations (NTC) were the highest for the article by Arnett DK (NTC = 9.37), followed by Bhatta (NTC = 4.54) [22], Wang JB (NTC = 3.36) [24], and Werner AK (NTC = 3.34) [25], showing that these papers have been rapidly gaining citations (Figure 7).

### 2.5. Keyword Structure, Thematic Landscape and Temporal Evolution

**Figure 8:**
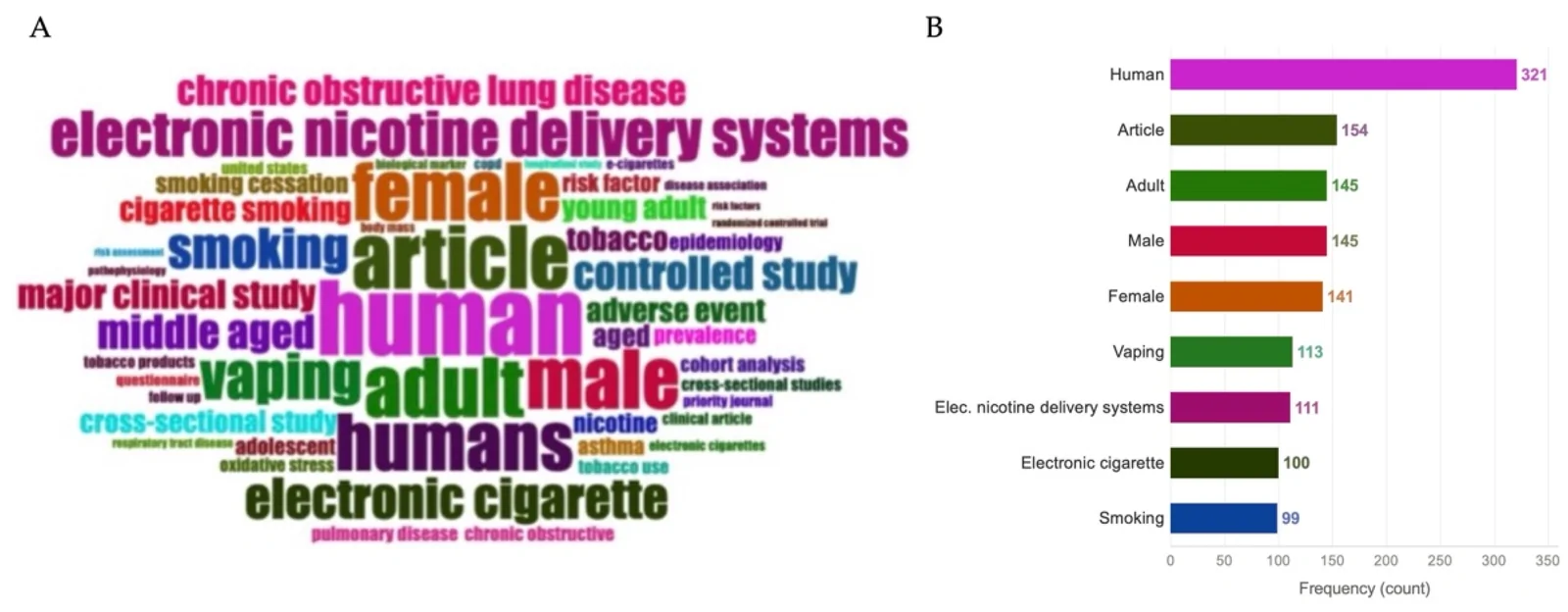
Keyword analysis of the OS-associated disease from vaping. (**A**) Word cloud; (**B**) frequency chart.

**Figure 9:**
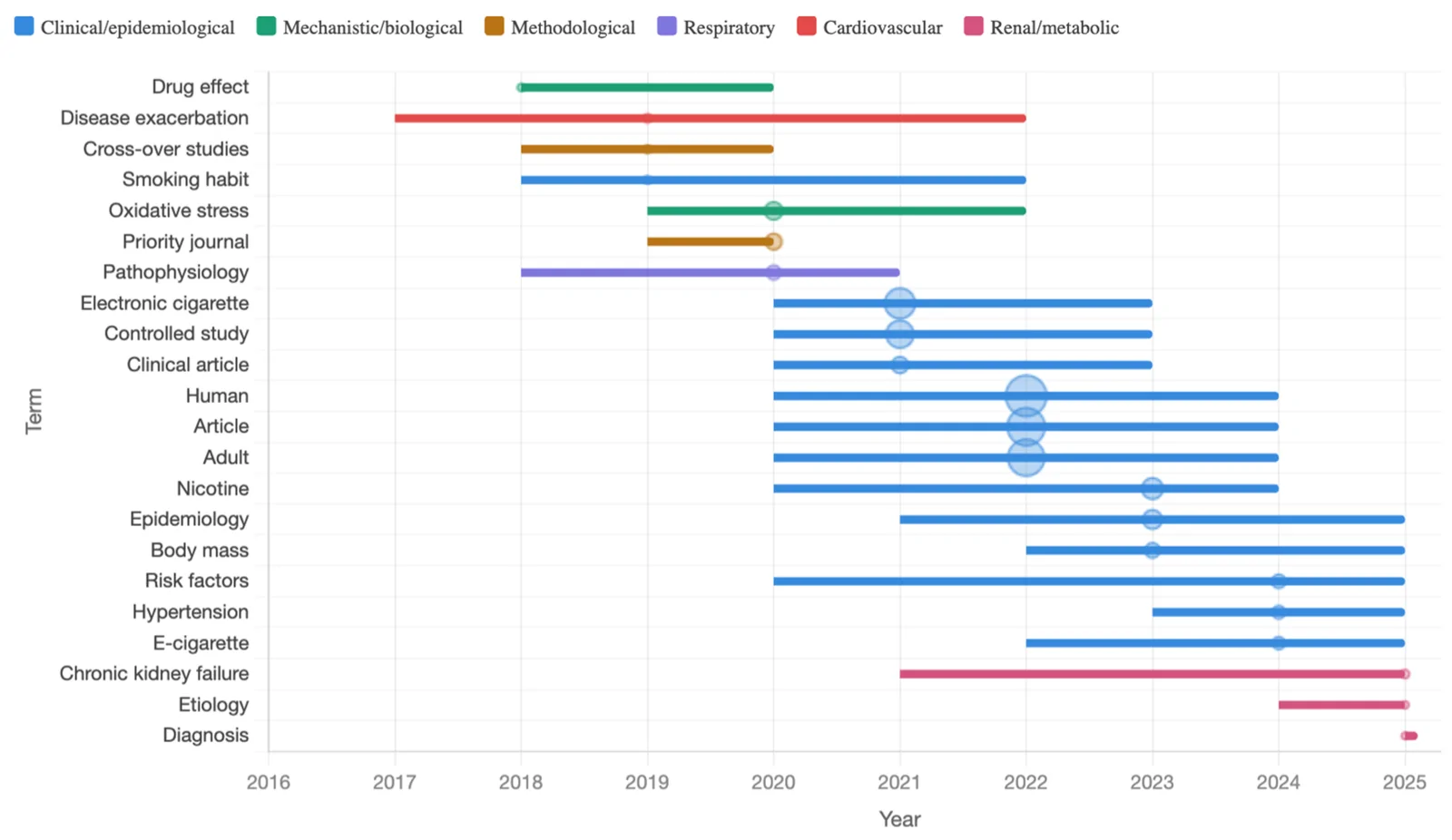
Trend topics over time (2016–2025). Bars represent Q1–Q3 IQR bars. Bubble sizes represent term frequency (smallest size = 5, intermediate size = 100, and largest size = 150).

Combined Figure 8 and Figure 9 present an overall well-structured and dynamic research landscape over time on vaping and electronic cigarettes in human populations organized into four groups. First, the keyword group surrounding EC products themselves is dominated by interchangeable terms such as electronic nicotine delivery systems, electronic cigarette, vaping, cigarette smoking, and tobacco product, which appear frequently throughout the entire study period (2016–2025). The second large group relates to the populations studied, including terms such as human, female, male, adult, adolescent, young adult, and middle-aged, with human (321 occurrences), adult, male, and female (145 each) being the most frequent in the word cloud (Figure 8A,B). This reflects the significant focus on age groups within populations, balanced with sex representation, in this research field. A third group of keywords encompasses those related to the types of studies employed in this research field, noted in prior articles (154 occurrences), including terms such as controlled study, cross-sectional study, major clinical study, questionnaire, and cohort analysis. This highlights a chronological evolution of observational studies in the earlier years of the study period toward more focused clinical and epidemiological studies as the field matured (Figure 9). The last group of keywords includes terms related to health outcomes, including chronic obstructive lung disease, pulmonary disease, chronic obstructive, adverse event, asthma, COPD, and oxidative stress. In the timeline/Gantt-style chart (Figure 9), the term oxidative stress retained the highest frequency with 34 occurrences in the three-year period 2019–2022, reflecting its position as the most prominent bridging theme within this corpus. Because oxidative stress was the primary search term used to build this study’s collection, its high frequency is expected. This result reflects our search strategy rather than providing an independent confirmation of the underlying biological mechanism. Figure 9 also shows a chronological “maturation” of the field with earlier research, from 2017 to 2020, centered on methodological aspects from exposure to drugs and nicotine, and acute effects on disease exacerbation, toward a broader spectrum of epidemiological studies oriented around the OS pathways. This shift concurred with the change in study types mentioned above. In other words, topics such as EVALI, nicotine dependence, and acute lung inflammation dominated in the early period up to 2019. After 2020, diseases related to the cardiovascular and renal systems and long-term exposure outcomes appeared in the timeline landscape. This new predominance of chronic disease in the research field indicates the emerging awareness among researchers that vaping injury may be more than solely limited to acute events such as EVALI and highlights the importance of developing diagnostic tools based on etiology.

**Figure 10:**
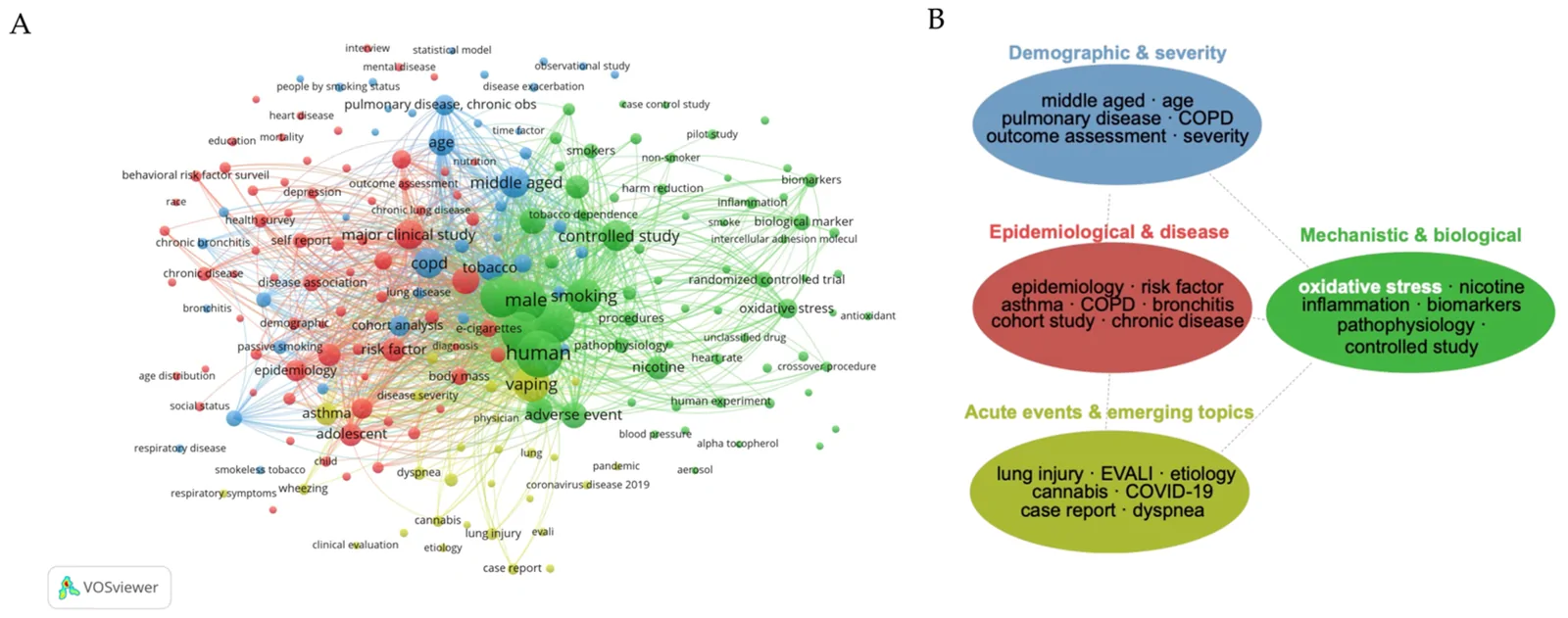
Keyword co-occurrence network. (**A**) Network map generated using VOSviewer; (**B**) cluster summary.

The keyword co-occurrence network (Figure 10) confirms the landscape patterns suggested by Figure 7 and Figure 8 to visualize OS-associated chronic disease from vaping in humans. In Figure 10A, the size of each circle represents the frequency at which a keyword appears, and the lines between circles show how often two keywords appear together in the same study. Based on these parameters, four main clusters have been identified and simplified from this complex network in Figure 10B. The blue cluster groups disease severity terms within the demographic cluster, with terms such as severity, outcome assessment, COPD, pulmonary disease, age, and middle-aged. It reflects the consistent trend of associating age groups within populations with the severity of vaping-associated lung disease. The red cluster encompasses epidemiological and disease-related terms such as epidemiology, risk factor, asthma, COPD, bronchitis, cohort study, and chronic disease, reflecting the systematic association between vaping exposure and pulmonary and systemic diseases at the population level. The largest and densest cluster is the green one, which is the most complex to interpret. It captures a combination of clinical terms such as pathophysiology, adverse event, biological markers, inflammation, oxidative stress, and includes central terms such as human, male, and smoking. Notably, oxidative stress appears as the most connected node within the green cluster, and the most connected of the four clusters identified. This position indicates that OS functions as a shared organizing framework across the clinical, epidemiological, and mechanistic literature. It should be interpreted as a description of the research landscape rather than independent evidence of a biological mechanism. Indeed, its peripheral location within the green cluster suggests that it is not restricted to a single specific pathway. Finally, the yellow cluster, the smallest and located at the bottom of the map, groups terms related to acute conditions of lung injury such as lung injury, etiology, cannabis, dyspnea, and EVALI, reflecting the early period of research focused on acute disease before the shift toward chronic disease outcomes, discussed in Figure 9.

Together, this analysis gives a comprehensive picture of the research field of OS-related disease from vaping that started with a narrow focus on acute lung injury and demographic description, with a progressive transition toward a broader, mechanistic approach based on the association of vaping exposure and chronic disease. Oxidative stress has emerged as the central core unifying these two dimensions. These findings reflect the growing global awareness among researchers, clinicians, and regulatory bodies that vaping-related harm is no longer restricted to acute events, and that OS represents a relevant, measurable target for future diagnostic and therapeutic strategies in this field.

For the next narrative sections, the literature published within the past five to ten years was identified through searches of PubMed and Scopus. Search strategies utilized combinations of the keywords “electronic cigarette”, “vaping”, “e-liquid”, “aerosol”, “oxidative stress”, “antioxidant”, and “chronic disease”. These strategies were supplemented by hand-searching the reference lists of key retrieved reviews for each component analyzed in Section 3 and Section 4. Studies were selected based on EC research addressing OS in human cohorts and in vitro human cell models. Both significant results and experimental regimens not reported in the original studies are presented in the subsequent sections.

## 3. Components of Electronic Cigarettes

**Figure 11:**
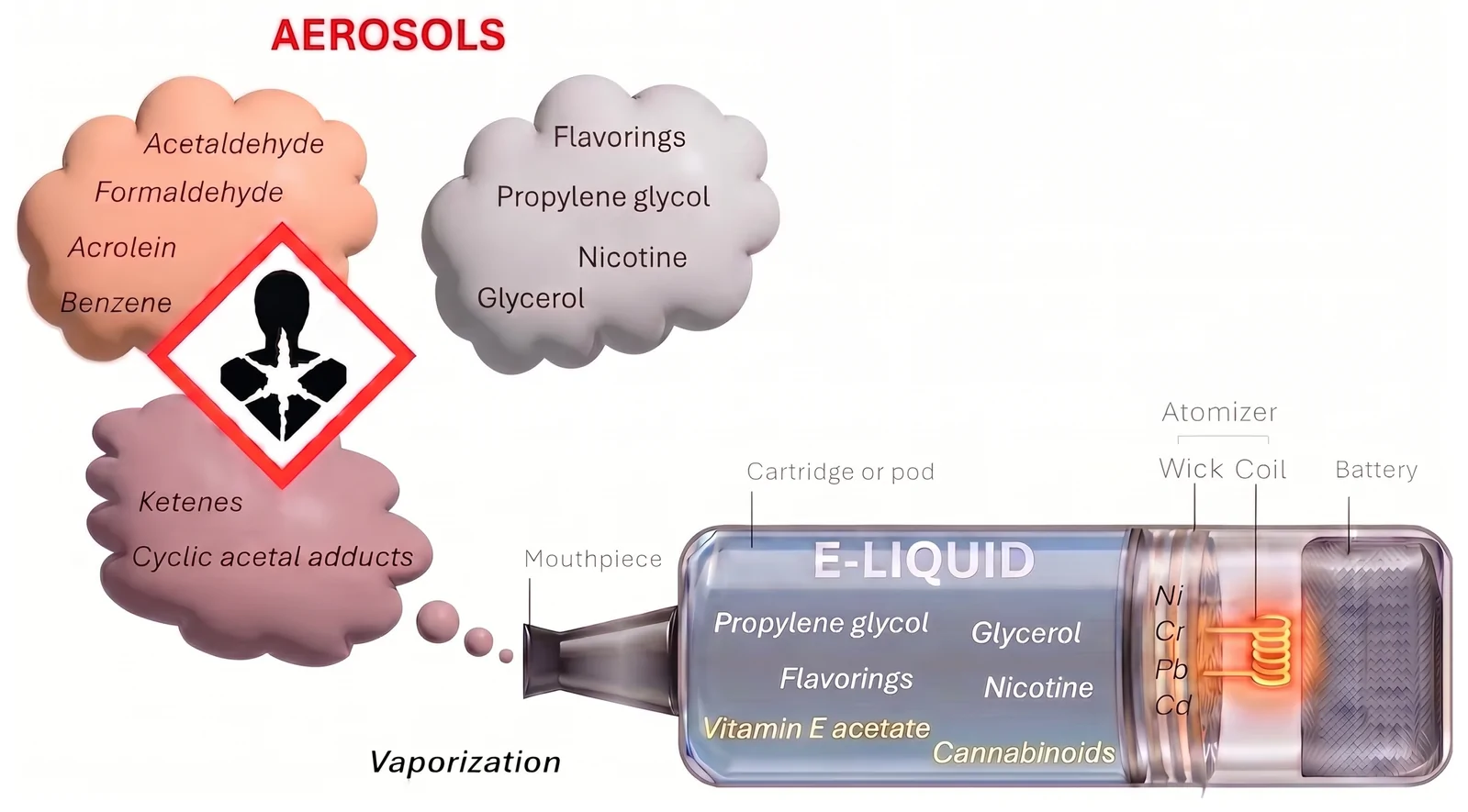
A simplified drawing that depicts aerosol compounds, contaminants, and potentially hazardous chemicals generated during the vaporization of e-liquid components, which occurs due to heating and thermal degradation initiated by the puffing process. The schematic distinguishes between unaltered evaporated carrier ingredients (gray cloud) and hazardous thermal degradation byproducts generated by heat (orange cloud), flagged with the GHS health-hazard pictogram to indicate that several of these byproducts carry individual regulatory hazard classifications. Non-nicotine and illicit additives (yellow text) present in the e-liquid generate pyrolytic emissions (brown cloud). Device-derived metallic contaminants that leach from the heating elements are nickel (Ni), chromium (Cr), cadmium (Cd), and lead (Pb).

For clarity, the constituents discussed below are grouped by origin, as illustrated in Figure 11: those added to the e-liquid, distinguishing standard components from non-nicotine products and illicit additives; and those found in the aerosol, either generated during heating and thermal degradation or introduced as device-derived metallic contaminants that leach from the coil and the wick.

### 3.1. E-Liquid Constituents, Non-Nicotine Products, and Illicit Additives

E-liquids are complex mixtures of glycerol (GL), propylene glycol (PG), nicotine, and flavorings, whose potential toxicity is driven by their thermal degradation products rather than their native chemical forms. These standard components are evaluated alongside non-nicotine vaping products containing cannabinoids and illicit additives like vitamin E acetate (VEA). This section reviews the characteristics and properties of these compounds and summarizes the most recent evidence regarding human health risks associated with chronic exposure.

#### 3.1.1. Alcohol-Derived Chemicals

The food industry uses PG and GL widely, as both are manufactured alcohol-derived chemicals used as thickeners, humectants, preservatives, and sweeteners owing to their hygroscopic properties. GL (glycerin; E422) is derived from vegetable oils and fats [31]. PG (1,2-propanediol; E1520), which has an acceptable dietary intake of 25 mg/kg body weight in adults [32], is manufactured from propylene oxide, a recognized carcinogen. Although the U.S. Food and Drug Administration (FDA) has classified both substances as “Generally Recognized as Safe” (GRAS) for food and pharmaceutical uses [20,33,34], their safety profile upon inhalation has never been formally established. Concentrations of GL and PG in commercially available e-liquids range from 0.3% to 98% of total composition, underscoring the potential inhalation exposure during vaping [34].

#### 3.1.2. Flavorings

Approximately 15,000 unique flavoring agents are currently used to enhance the appeal of e-liquids [8,35,36]. While these substances are deemed safe when ingested as food additives, compounds such as diacetyl (2,3-butanedione) and pentanedione raise concerns upon chronic inhalation, particularly in adolescents [37]. An additional layer of complexity arises from the thermal decomposition of flavorings during vaporization, which generates byproducts of unknown toxicity at undefined safe limit concentrations [35]. Aldehydic flavorings, including vanillin, berry, and fruit compounds, undergo chemical reactions with PG and GL to form cytotoxic cyclic acetal adducts detected in EC aerosols [38,39,40]. Several countries, including Australia, have enacted restrictive regulations limiting tobacco flavorings in e-liquids to discourage vaping among non-smokers, though such bans risk driving users back to conventional tobacco products [41,42].

#### 3.1.3. Nicotine

Nicotine is a highly addictive natural alkaloid present in many, though not all, e-liquids. Reported concentrations ranging from 0 to 100 mg/mL vary widely, and permitted maxima differ by jurisdiction, such as the 20 mg/mL limit in the European Union, with less than 1% of products being genuinely nicotine-free [43]. During vaping, the transformation of nicotine into salts by its protonation with benzoate produces byproducts that are more efficiently absorbed into the bloodstream, increasing the risk of potential addiction [44]. A growing body of evidence challenges the cessation rationale for ECs, suggesting that these devices may drive rather than attenuate nicotine dependence [45,46,47]. Nicotine acts on the dopaminergic reward circuit and may facilitate addiction to other substances via epigenetic mechanisms [48] or maladaptive neuroplasticity. Paradoxically, reducing nicotine concentrations in e-liquid prompts longer and more frequent puffing, increasing user exposure to carbonyl compounds [49]. In response to the public health concerns posed by flavored nicotine products, the World Health Organization has called for an urgent ban, and fifty countries have already implemented such measures [50].

#### 3.1.4. Non-Nicotine Vaping Products: Cannabinoids

Cannabis-derived substances used in e-liquids include the non-psychoactive cannabidiol (CBD)—approved for selected neurological indications—and the psychoactive Δ9-tetrahydrocannabinol (THC), which remains illegal in many jurisdictions. In e-liquids, CBD is dissolved in PG:GL mixtures; however, thermal conversion of CBD to THC during vaping has been demonstrated and shown to depend on coil temperature, solvent ratio, and vaping frequency [51,52,53]. The absence of transparent and accurate CBD labeling on EC products reinforces concerns that these devices can function as vehicles for illicit drug delivery [54], particularly as recreational cannabis markets expand [55].

#### 3.1.5. Illicit Additives and Adulterants: Vitamin E Acetate

Vitamin E acetate (VEA; alpha-tocopheryl acetate), a thickening agent used in dietary supplements, food additives, and skin care products, is illicitly added to cannabis vape products [56,57]. Despite its antioxidant reputation in nutritional contexts, VEA has been directly implicated in the outbreak of EC-associated acute lung injury (EVALI) [58,59]. The National Institute on Drug Abuse (NIDA) and the FDA have issued national alerts recommending that individuals avoid any product containing VEA or THC obtained from informal sources [60]. As with cannabinoids, VEA contamination is largely confined to informal or illicit supply chains, but because there is no comprehensive market control, these separate product categories often mix on the unstandardized vaping market [56].

### 3.2. Aerosol By-Products and Device-Derived Contaminants

Although EC aerosols are not chemically equivalent to cigarette smoke, they are nonetheless potentially harmful. Produced through the heating and vaporization of e-liquid, aerosols contain all constituents of the e-liquid—PG, GL, flavorings, and nicotine—alongside byproducts generated during heating and thermal degradation, including aldehydes, psychoactive substances, and benzene (Figure 11). The wicking material (cotton, ceramic, or silica) further contributes to aerosol heterogeneity by emitting different compounds depending on heat resistance and chemical stability [61]. The coil and wick additionally release metallic contaminants into the aerosol.

#### 3.2.1. Aldehydes

Acrolein is a highly toxic aldehyde found in traditional cigarette smoke [62] and is also detected in EC aerosols at concentrations exceeding those in tobacco smoke under high-power or dry-puff conditions, whereas under normal-use conditions, reported yields are generally lower than in traditional cigarettes [53]. As few as fifteen puffs can emit 3–61.5  μg of acrolein—a potent stimulator of respiratory distress and cardiovascular disease [63]. Formed during the oxidation of PG and GL, acrolein is an important contributor to EC-related lung toxicity, asthma exacerbation, and lung cancer risk [64]. Formaldehyde and acetaldehyde are produced in quantities that exceed the lowest-observed-adverse-effect level for respiratory harm during chronic vaping [49,53]. Regarding its carcinogenic status, acetaldehyde is classified by IARC (International Agency for Research on Cancer) as a Group 1 human carcinogen in the context of alcoholic-beverage consumption, while as an isolated chemical agent, it has historically been evaluated separately as Group 2B [65]. These yields depend strongly on coil temperature, device power, and puff topography. Comparative human data indicate that toxicant exposure may be lower in EC users than in combustible-cigarette smokers, although some inflammatory effects may be comparable [66,67].

#### 3.2.2. Benzene

Benzene, a potent volatile aromatic carcinogen [68], is formed during the pyrolysis of PG and GL. A recent analysis of 43 e-liquids and aerosol samples confirmed the consistent presence of benzene and ethylbenzene at quantifiable concentrations [57]. Reported benzene levels are markedly lower than in traditional-cigarette smoke and increase with device power. This indicates that benzene formation depends on exposure conditions rather than being intrinsic to EC use [57].

#### 3.2.3. Ketenes

Ketenes, highly reactive carbon–carbon compounds, arise from the pyrolysis of acetate forms, principally VEA, during vaping. Metal components of the wick catalyze this thermal transformation, and the resulting ketenes are inhaled by both EC users and bystanders [69]. While classified among the most toxic emissions identified to date in EC aerosols, VEA-derived ketenes represent a significant risk factor for acute lung injury [70]. However, ketene formation has been demonstrated principally with acetate-containing additives such as VEA and has not been reported at comparable levels in standard nicotine vaping.

#### 3.2.4. Cyclic Acetal Adducts

E-liquids contain diverse chemicals capable of interacting to form novel cyclic acetal adducts. These arise primarily through condensation reactions between the carrier solvents (PG and GL) and aldehyde flavorings, yielding cytotoxic compounds exhaled as aerosols and potentially introducing localized environmental exposure pathways for users and bystanders [71,72,73]. Cinnamaldehyde and benzaldehyde—despite not being listed as EC constituents—have demonstrated cytotoxic effects on the respiratory epithelium [74,75]. Additionally, acrolein-derived DNA adducts (α- and γ-OH-Acr-dG) are potentially carcinogenic and have recently been implicated in the early carcinogenic transformation of buccal cells [76,77].

#### 3.2.5. Metal Elements

A wide array of metals—including arsenic, chromium, aluminum, selenium, iron, lead, titanium, cobalt, molybdenum, nickel, and manganese—has been identified in EC cartridges, tanks, and wicks [78]. Chromium and lead are classified as probable carcinogens by both the US Environmental Protection Agency and the IARC. Systematic review data indicate that most EC-derived metals are absorbed and detectable in saliva, urine, and serum at higher concentrations than those associated with conventional cigarette use [78]. Corroborating these findings, a Korean cohort confirmed elevated serum lead, cadmium, and mercury in EC users compared to traditional smokers, which was attributable to coil leaching during heating [79]. Certain metals, particularly chromium and aluminum, further catalyze the formation of cytotoxic acetals and aldehydes from PG [72]. The presence and release of these device-derived contaminants depend heavily on device type, wattage settings, and coil aging; therefore, the inhalation risk is a function of specific exposure conditions rather than the absolute presence of these elements in the hardware [53,78].

## 4. From Aerosols to Disease: Oxidative Stress Mechanisms, Endogenous Antioxidant Defense Responses, and Translational Implications of EC Toxic Components

### 4.1. Oxidative Stress Mechanism of EC Toxic Components

Inadequate quality control, inaccurate labeling, the enormous diversity of flavoring agents, and variability in coil and wick composition collectively impede the establishment of global safety standards [80]. Despite mounting evidence of health risks linked to vaping, the toxic components of e-liquids and e-liquid-derived aerosols remain subject to evolving regulation in several jurisdictions, such as the US FDA’s Premarket Tobacco Product Application (PMTA) and the EU tobacco products directive. However, rather than a single missing standard, three distinct issues coexist: first, the absence of dedicated legislation in some jurisdictions; second, the heterogeneity of existing regulatory frameworks; and third, the lack of harmonization at the global level, which together leave vapers and bystanders exposed to incompletely characterized risks.

**Figure 12:**
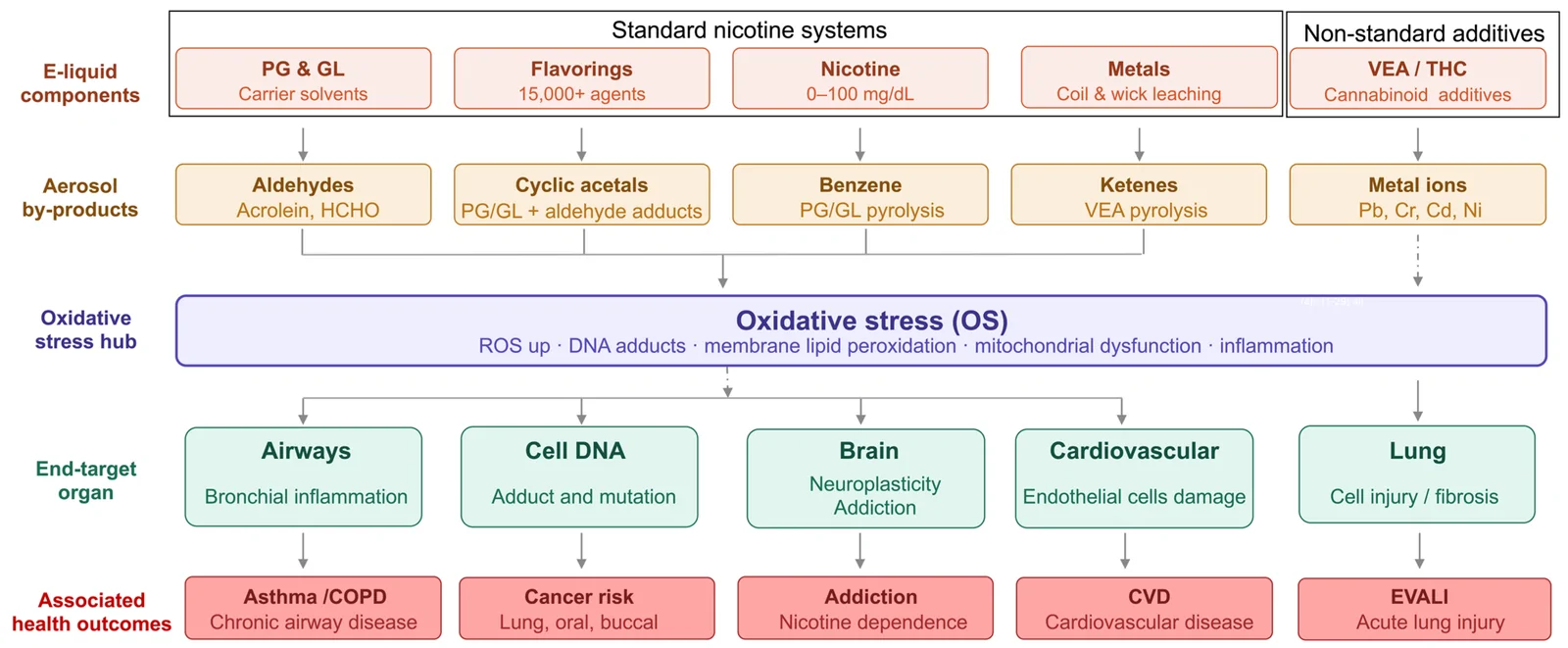
The schematic suggests how thermal degradation of e-liquid chemicals generates distinct aerosol byproducts and contaminants converging on oxidative stress (OS). The category of non-standard additives (VEA/THC), which encompasses non-nicotine products and illicit adulterants, is structurally distinguished from standard nicotine systems. Downstream cellular responses propagated through the OS hub affect specific end-target organs, driving associated health outcomes across respiratory, nervous, and cardiovascular systems, as well as cellular DNA injuries. Abbreviations: PG, propylene glycol; GL, glycerol; HCHO, formaldehyde; EVALI, e-cigarette or vaping product use-associated lung injury; VEA, vitamin E acetate; THC, tetrahydrocannabinol; Pb, lead; Cr, chromium; Cd, cadmium; Ni, nickel; COPD, chronic obstructive pulmonary disease; ROS, reactive oxygen species; DNA, deoxyribonucleic acid; CVD, cardiovascular disease.

Although the preclinical studies underlying the mechanisms presented in Table 2 and Figure 12 vary considerably depending on the device, power, coil, wick composition, puff topography, e-liquid composition, exposure system, and dose normalization, the overall pattern remains consistent. Indeed, virtually every class of e-liquid-derived aerosol byproducts, including aldehydes, aromatic hydrocarbons, heavy metals, flavorings, and cannabis additives, converges on oxidative stress through diverse mechanisms. Specifically, cellular membrane lipid peroxidation is driven by acrolein, mitochondrial dysfunction is induced by diacetyl, ROS-mediated DNA strand breaks are driven by benzene, and glutathione depletion is caused by coil-leached heavy metals. However, while standardized protocols (such as ISO 20768) exist to govern mechanical aerosol generation and puffing regimens, these standards do not extend to biological in vitro exposure design. As shown in Table 2, the available preclinical studies largely do not specify a standardized puffing regimen, nor do they report dry-puff verification, air-liquid interface configuration, or dose normalization for in vitro exposure models. This heterogeneity limits direct quantitative comparison across studies, though not qualitative comparison. Notably, several of these attributions concerning compounds such as formaldehyde, acetaldehyde, benzene, ketenes, and chromium or nickel rely on general emission data or inference from non-EC research rather than on EC-specific focused research models. Therefore, these results should be interpreted as converging lines of evidence of differing strengths instead of directly comparable quantitative findings.

### 4.2. Alteration of the Antioxidant Defense Mechanisms by Exposure to EC Components

The key mechanism by which the human body responds to oxidative stress is mostly driven by the transcription factor NRF2 (nuclear factor erythroid 2-related factor 2), which is normally kept inactive in the cytoplasm by the regulatory protein KEAP1 (Kelch-like ECH-associated protein 1) [81,82]. Upon exposure to acrolein or heavy metals that modify KEAP1, NRF2 is released and translocated to the nucleus, where it promotes the transcription of antioxidant enzymes such as HO-1 (heme oxygenase-1) and NQO1 (NADPH quinone oxidoreductase 1).

Human studies of EC users report reduced superoxide dismutase (SOD) and catalase below the levels observed in non-smokers, a reversal of the glutathione redox ratio (GSH:GSSG), and an upregulated NOX-2-derived ROS appearance, consequently accelerating ROS accumulation [67,83]. Read together with the NRF2/KEAP1 mechanism characterized in other chronic-exposure settings [81,82], these observations are compatible with a biphasic NRF2 mechanism, featuring an adaptive response to short-term exposure to acrolein, aldehydes, and heavy metals that leads to NRF2 activation, triggering an initial protective antioxidant response. The second phase would be characterized by progressive NRF2 exhaustion due to sustained and chronic exposure overwhelming the antioxidant defense mechanisms. This NRF2 exhaustion is suggested to prompt a transcriptional switch that downregulates antioxidant enzymes involved in the detoxification and elimination of reactive oxygen species (ROS).

**Figure 13:**
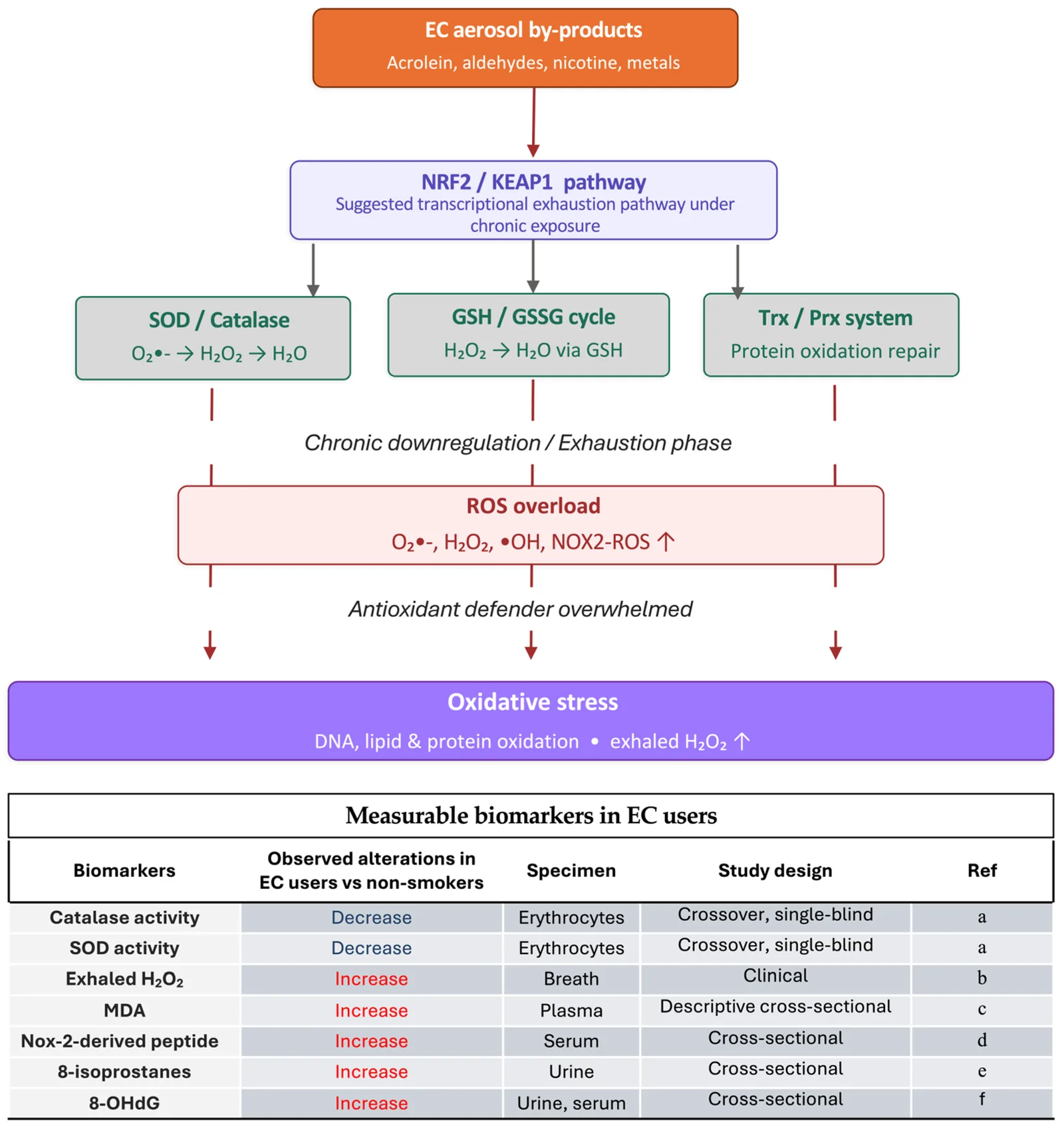
Proposed framework for NRF2 pathway exhaustion under e-cigarette (EC) aerosol exposure. Chronic exposure to aerosol byproducts is hypothesized to parallel a biphasic shift from initial endogenous antioxidant defense activation to possible transcriptional downregulation of the NRF2/KEAP1 master regulatory pathway and its downstream enzymatic defenses—superoxide dismutase (SOD)/catalase, the glutathione peroxidase (GPx)/glutathione (GSH) cycle, and the thioredoxin (Trx)/peroxiredoxin (Prx) system. This exhaustion is associated with reactive oxygen species (ROS) overload and measurable oxidative stress across specific human cohorts. Biomarker changes observed in human EC users are indicated vs. non-smoking controls. Letters in the righ-most column correspond to studies in the literature: a, [83]; b, [87]; c, [84]; d, [28]; e, [86]; f, [20,86,88].

In humans, controlled and observational studies have linked EC use to endothelial cell dysfunction and vascular oxidative stress, including reduced flow-mediated vasodilation and elevated markers of oxidative injury and inflammation. A three-period randomized crossover trial conducted in tobacco smokers reported that vaping with nicotine impaired microvascular vasodilation and increased arterial stiffness and OS, whereas neither sham vaping nor vaping of the PG-GL vehicle alone modified these parameters [26]. Furthermore, an open-label, randomized, crossover study showed that smokers acutely exposed to ECs with nicotine exhibited disruption of the autonomic nervous system by a shift toward sympathetic dominance and a compromised antioxidant defense compared to vaping without nicotine [27]. The SUR-VAPES 2 randomized trial found that acute EC exposure raised soluble NOX-2-derived peptide and reduced flow-mediated dilation, although to a lesser extent than that observed in traditional tobacco smokers [28]. A recent systematic review and narrative synthesis integrated this clinical evidence, linking exposure to ECs and heated-tobacco products to the activation of OS and to the early onset of atherosclerotic and cardiovascular alterations in humans [84]. Specifically, the overproduction of ROS can be clinically monitored through distinct specimens containing excreted biomarkers, allowing the tracking of endothelial and macrovascular alterations (Figure 13). Human clinical evidence outlines that the upregulation of NOX-2-derived peptides directly mirrors the systemic oxidative burden that triggers early vascular damage. This pro-oxidant state induces lipid peroxidation and DNA damage, which are captured by elevated concentrations of plasma malondialdehyde (MDA), urinary 8-isoprostanes, and serum or urinary 8-hydroxydeoxyguanosine (8-OHdG). Nevertheless, it is important to consider that these controlled human exposures indicate nicotine as the main driver of the acute vascular and oxidative response. In contrast, the carrier solvents, flavorings, and device-derived metals rely largely on in vitro human cell models, as presented in Table 2, and have not yet been confirmed in controlled human protocols. Beyond these acute effects, the transition from acute adaptation to chronic antioxidant insufficiency may explain the progressive organ damage observed in chronic disease onset, as shown in Figure 12. This underscores that vaping injury is not solely restricted to acute events but instead increases over time through the “NRF2 exhaustion model”. Because these studies documenting OS and toxicant exposure in EC users do not provide direct human evidence for the complete NRF2/KEAP1 exhaustion sequence, the framework illustrated in Figure 13 is therefore presented as a suggestive synthetic model of converging mechanisms rather than a strict established evidence strength.

Although these chronic human cohort studies converge toward the same conclusion of OS-associated chronic disease, their interpretation requires caution. Most participants, classified as EC users, were former smokers, and several biomarkers in EC users fell between those of current smokers and non-users instead of being exclusively linked to EC use [85]. Conversely, longitudinal data from Wave 1 of the PATH (Population Assessment of Tobacco and Health) study cohort showed that adult former smokers who switched exclusively to ECs had levels of biomarkers of inflammation and OS, including urinary F_2_-isoprostane, similar to those of former smokers who had stopped all tobacco use, and to those of the never-users [86]. Smoking history and time since cessation are important covariates in this literature, discriminating between acute and chronic exposure.

### 4.3. Biomarkers to Assess Disruption of Antioxidant Defense Associated with EC Exposure

Beyond the NRF2/KEAP1 pathway, several other antioxidant defense molecules are targeted by the toxic EC components during oxidative damage. These include 8-hydroxydeoxyguanosine (8-OHdG), which reflects the oxidative damage of nuclear DNA; malondialdehyde (MDA) and 8-isoprostanes, which indicate lipid peroxidation; and hydrogen peroxide (H_2_O_2_), which reveals systemic oxidation. All these biomarkers, measurable from accessible specimens such as blood [83], plasma [85], serum [87], urine [21,67,88], and exhaled breath [66], can provide only an estimate of the OS burden in EC users (Figure 13) as no thresholds have been designated.

Together, these data from this narrative review underscore that the transformation of EC chemical components into byproduct aerosols raises concerns that are not yet fully addressed by current safety regulations. Furthermore, chronic exposure to these toxic aerosols for both EC users and bystanders carries a long-term disease risk that remains to be fully quantified.

### 4.4. Convergence of Data from Combined Bibliometric Analysis and Narrative Review and Future Perspectives

The bibliometric analysis and narrative review align conceptually in highlighting OS as the central and bridging theme linking EC exposure to chronic disease. This relationship is evaluated through different forms of evidence. However, because OS was utilized as a baseline search term, this structural alignment maps a shared academic focus rather than demonstrating an independent finding.

As described in Section 3, EC aerosol components trigger OS through direct ROS generation and progressive NRF2/KEAP1 exhaustion, shifting the antioxidant defense from an initial protective response to a chronic antioxidant defense deficit that worsens with sustained exposure duration. This timing directly mirrors the chronological pattern observed in Figure 9, in which earlier studies (2017–2020) focused on acute events such as EVALI and short-term exposure, while after the outbreak, the research landscape shifted toward chronic disease targeting the cardiovascular and renal systems. This reveals a striking thematic convergence between global literature trends and molecular toxicity mechanisms, in which the chronological maturation observed in the bibliometric timeline parallels the expanding academic focus on the hypothesized NRF2 biphasic framework. Indeed, early cross-sectional studies captured mostly short-term vapers whose initial adaptive response was asymptomatic, while the chronic antioxidant system deficiency and progressive cellular insufficiency become detectable as organs become the end-targets of the long-duration EC exposure. As researchers and clinicians have progressively investigated NRF2, the chronology of these scientific advances has aligned with the conceptual stages of the “NRF2 biphasic response”. To synthesize this cross-disciplinary mapping effectively, it is necessary to distinguish between the bibliometric body of literature that is strictly limited to human observational studies, and the molecular signaling pathways heavily relying on in vitro human cell models presented in the narrative review. These distinct levels of evidence, although fundamentally complementary, are not interchangeable. Thus, any extrapolation from preclinical protocols to human clinical applications must be made with caution. Our data track a clear structural shift in publication trends over the last decade, developing across three observable intervals. An initial exploratory phase occurred between 2016 and 2018, characterized by limited publication activity. This baseline shifted into a rapid expansion phase from 2019 to 2022, marked by substantial increases in annual output and growth rates. Most recently, the 2023–2025 window indicates a period of academic consolidation during which publication productivity stabilized at a relatively high level while cumulative scientific knowledge continued to grow. These structural trends confirm that vaping-associated OS has become an established area of research with sustained international scientific interest.

Convergence is further confirmed by the keyword co-occurrence analysis presented in Figure 10, where OS is revealed as the most connected node across all four clusters, bridging epidemiological, clinical and mechanistic research. Strikingly, the key finding of this combined analysis is that OS is the main biological mechanism explored in the narrative review and the most frequent bridging keyword in the bibliometric research landscape. These two different perspectives, one mechanistic and one bibliometric, align to reflect a common research focus from which scientific communities across disciplines and study designs have independently and progressively identified OS as an important factor in EC-associated chronic disease.

The biomarkers described in Figure 13 translate this mechanistic evidence into measurable parameters across noninvasive specimens. Their integration into future longitudinal studies could address the critical gap identified in this combined analysis: the absence of dose–response data and exposure thresholds across sexes, age groups and device types for e-liquid components and aerosols. Moving forward, future research agendas must prioritize three areas: (1) standardizing air-liquid interface preclinical protocols incorporating device power, coil and wick materials, puff topography, and dose normalization, to enhance study reliability and comparability; (2) executing long-term human cohort studies to monitor and track exposure thresholds across age, sex, and device types using non-invasive biomarkers like urine, exhaled breath, and saliva; and (3) separately studying regular nicotine ECs and illicit black-market cannabinoids to distinguish their health impact. These data are urgently needed to provide a regulatory framework and justify the control of EC component composition and emission standards.

Together, this bibliometric analysis and narrative review indicate that the field of OS-related chronic diseases from vaping has matured during the last decade from a narrow focus on acute lung injury toward a mechanistic, multidisciplinary understanding to guide future diagnostic, therapeutic, and regulatory strategies.

### 4.5. Limitations

Several limitations of this integrated review merit acknowledgment. The narrative component is necessarily selective; the EC literature is vast and growing, and no review can claim to capture every relevant study. The bibliometric analysis was based exclusively on the Scopus database, which does not index all published literature, and a single search date (20 March 2026), introducing indexing-dependent selection effects. Studies published in languages other than English were systematically excluded. A significant limitation involved the bibliometric search itself because “oxidative stress” was included as a baseline search term to meet the objective of the study. This has led to a high frequency and high network centrality, which should be cautiously interpreted solely within the context of the bibliometric dataset of this review; thus, OS should not be interpreted as an independent validation of the biological mechanism linking ECs to chronic disease. As noted earlier, the deliberate choice to rely solely on the Scopus database was motivated by its broader coverage, multidisciplinary scope, and built-in citation tracking over Web of Science and PubMed. While merging these three databases would have made our study more rigorous and prevented coverage-dependent effects, Scopus was deemed sufficient for the scope of this analysis. Another limitation lies in the divergence between the bibliometric analysis mapping human clinical and epidemiological data, and the narrative review, which intentionally selected the most recent preclinical in vitro human models, necessary for explaining signaling pathways. The exclusion of animal studies was a deliberate choice to maximize clinical and epidemiological data. This implies that the present study omits mechanistic evidence that has not yet been validated in human populations. Finally, bibliometric co-occurrence networks visualize topical proximity but do not establish the directionality, quality, or causal weight of associations between the two research themes.

### 4.6. Research Gap and Future Directions

Our bibliometric analysis identifies four gaps that the narrative review cannot close on its own. First, the very low multi-country publication fraction, together with a concentration of the ten leading institutions in the US, highlights the need for coordinated international consortia and harmonized reporting to foster a global perspective on universal standards. Second, since 2020, research has frequently linked heart and kidney health risks together, yet the corresponding mechanistic evidence is missing. Future research must therefore prioritize how OS damages the kidneys and the vasculature upon EC exposure. Third, the acute events of the EVALI outbreak stand out as a distinct pattern driven by the illicit use of VEA as an adulterant in unregulated THC vaping products, separate from daily EC use. This distinction prevents these separate products categories from being wrongly conflated with the toxicological profiles of regulated nicotine products. Fourth, current human research lacks critical evidence on how vape dose, device type, and frequency of use determine long-term exposure thresholds and clinical outcomes. To resolve this data gap, long-term monitoring studies, through international collaborations, should track real-world users over time, stratified by age, sex, and the specific devices and products they use, using non-invasive OS biomarkers measured in exhaled breath or urine. Together, these approaches would improve the comparability on which such thresholds depend.

## 5. Conclusions

The significance of this review lies in its combined approach, as it is the first to combine a bibliometric analysis and a narrative synthesis to provide an integrated picture of how EC exposure has been associated with chronic disease through OS-related pathways. Although electronic cigarettes were initially introduced as a promising cessation tool for nicotine-dependent smokers, our findings indicate that this original promise has been complicated by the rapid increase in EC use among never-smokers and adolescents and by unresolved toxicological concerns. The bibliometric analysis describes a research field that has grown rapidly over the last decade, progressively shifting from early, acute-centered research toward a broader focus on chronic disease outcomes; within this corpus, oxidative stress was the most frequent bridging keyword and the most connected theme in the global research landscape; however, this prominence does not independently confirm the mechanism. In turn, the narrative synthesis indicates that ECs deliver a diverse and incompletely characterized mixture of components that have been associated with markers of OS across multiple body systems, particularly the cardiovascular, pulmonary, digestive, and immune systems. These conclusions rest on a selective synthesis and do not establish causation. Furthermore, our findings highlight geographical inequities and a disciplinary gap between epidemiology and mechanistic research around oxidative stress. By addressing the “what”, “who”, “where”, “how”, and “how much” of OS-associated chronic disease from vaping, this review provides researchers with a clearer and more comprehensive picture of the current state of the science on this emerging public health concern. Ultimately, it offers clinicians a synthesis of the most relevant disease mechanisms proposed to link vaping-associated oxidative stress to chronic disease and equips policymakers with an evidence-based framework to identify the regulatory gaps necessary for an effective response to the rapid global increase in EC use.

## Figures and Tables

**Table 1:** Top 10 Most Relevant Affiliations.

Rank	Affiliation	Articles
1	University of California	8
2	Roswell Park Comprehensive Cancer Center	6
3	New York University	6
4	Florida International University	6
5	David Geffen School of Medicine (UCLA)	5
6	Geisel School of Medicine at Dartmouth	5
7	National Center for Environmental Health (CDC)	5
8	Center for Tobacco Products (FDA)	5
9	University of Catania	5
10	Johns Hopkins University School of Medicine	4

**Table 2:** Vaping-associated oxidative stress: Toxic components, mechanisms and disease outcomes.

Toxic By-Product	Chemical Reaction on E-Liquid Substrate	Oxidative Stress Mechanism	Associated Disease	Evidence Base and Reporting Gaps	Ref.
ALDEHYDES and CARBONYLS					
Acrolein	Oxidation of PG and GL	Lipid peroxidation; respiratory epithelial ROS; mitochondrial stress	Lung toxicity, asthma exacerbation, lung cancer	In vitro, not ISO-specified; dry-puff NR	[62,63,64]
Formaldehyde	Thermal degradation of PG and GL	DNA adduct formation; mucosal oxidative inflammation	Respiratory harm; Probable carcinogenesis	Emissions only; no cellular model	[49,53]
Acetaldehyde	Thermal degradation of PG and GL	Protein carbonylation; DNA adduct formation; mitochondrial dysfunction	Probable carcinogenesis; chronic airway injury	Emissions only; no cellular model	[49,53,65]
AROMATIC HYDROCARBONS					
Benzene	Pyrolysis of PG and GL	ROS via epoxide metabolites; DNA strand breaks; bone marrow toxicity	Hematological cancers; lung cancer risk	Emissions only; no cellular model	[57,68]
CYCLIC ACETAL ADDUCTS					
Cyclic acetals (PG/GL; aldehydes)	Condensation of PG/GL with aldehyde flavorings	Cytotoxicity to respiratory epithelium; DNA adduct formation	Airway inflammation; oral/buccal carcinogenesis	In vitro, dose normalization NR	[38,71,72,73]
Acrolein-DNA adducts	Acrolein reaction with guanine bases in DNA (Michael reaction)	DNA adduct formation	Early carcinogenic transformation of buccal cells	Human biomonitoring	[76,77]
Cinnamaldehyde/Benzaldehyde	Thermal decomposition of flavoring agents	Electrophilic attack on respiratory epithelium; thiol depletion	Cytotoxicity; airway epithelial injury	In vitro, dry-puff NR	[74,75]
CANNABIS ADDITIVES					
Vitamin E Acetate (VEA)	Ketene formation	Cellular lipid membrane disruption; surfactant layer damage	EVALI (e-cigarette/vaping-associated lung injury)	In vitro/simulation; clinical case series	[56,58,59,60]
Ketenes (VEA-derived)	Metal-catalyzed pyrolysis of VEA during vaping	Highly reactive oxidants; rapid protein and lipid alkylation	Acute lung injury; secondhand bystander exposure	Emissions only; no cellular model	[69,70]
HEAVY METALS					
Lead (Pb), Cadmium (Cd)	Coil/wick leaching during heating	Redox cycling; mitochondrial ROS; Glutathione depletion; catalysis of aldehyde formation	Systemic toxicity; probable carcinogenesis; cardiovascular harm	Human biomonitoring; mechanism inferred	[78,79]
Chromium (Cr), Nickel (Ni)	Coil/wick leaching during heating	Cr (VI) oxidative DNA damage; Ni epigenetic ROS dysregulation; catalysis of acetals	Lung carcinogenesis (IARC probable); endothelial damage	Emissions only; no cellular model	[72,78,79]
NICOTINE and NEUROACTIVE					
Nicotine benzoate	Protonation (Nicotine Salt Formation)	Dopaminergic ROS; epigenetic dysregulation; maladaptive neuroplasticity	Nicotine dependence; cross-addiction; cardiovascular oxidative harm	Human studies; OS mechanism inferred	[44,45,46,48]
Nicotine	Principal e-liquid alkaloid (0–100 mg/mL)	Dopaminergic ROS; epigenetic dysregulation; maladaptive neuroplasticity	Nicotine dependence; cross-addiction; cardiovascular oxidative harm	Human controlled exposure; OS mechanism inferred	[45,46,48]
FLAVORING AGENTS					
Diacetyl/ Pentanedione	Butter Cream flavoring agents	Bronchiolar oxidative inflammation; mitochondrial dysfunction in club cells	Obliterative bronchiolitis (‘popcorn lung’); irreversible airway fibrosis	Occupational epidemiology; EC evidence limited	[37]

Abbreviations: ROS, reactive oxygen species; PG, propylene glycol; GL, glycerol; VEA, vitamin E acetate; EVALI, e-cigarette/vaping-associated lung injury; DNA, deoxyribonucleic acid; EC, electronic cigarette; ISO, International Organization for Standardization; NR, not reported.

## Data Availability

The original data presented in the study are openly available in the Open Source Framework repository at https://osf.io/kpgq5/overview?view_only=615b70d3b02a4b14a7e859398589695c (accessed on 9 July 2026).

## Abbreviations

CBD	Cannabidiol
COPD	Chronic obstructive pulmonary disease
EC	Electronic cigarette
EVALI	E-cigarette or vaping product use–associated lung injury
FDA	Food and Drug Administration (U.S.)
GL	Glycerol
OS	Oxidative stress
PG	Propylene glycol
ROS	Reactive oxygen species
THC	delta-9-tetrahydrocannabinol
VEA	Vitamin E acetate
